# Immunostimulation of *Parasteatoda tepidariorum* (Araneae: Theridiidae) in juvenile and adult stages. Immunity reactions to injury with foreign body and *Bacillus subtilis* infection

**DOI:** 10.7717/peerj.15337

**Published:** 2023-07-17

**Authors:** Agnieszka Ewa Czerwonka, Marta Katarzyna Sawadro, Jolanta Brożek, Agnieszka Izabela Babczyńska

**Affiliations:** University of Silesia, Katowice, Poland

**Keywords:** Immunocompetence, Invertebrate immunity, Immunostimulants, Bacterial infection, Injury, Spider, *Parasteatoda tepidariorum*, Immunostimulation

## Abstract

To assess the immune potential of spiders, in the present study juvenile and adult females of *Parasteatoda tepidariorum* were exposed to *Bacillus subtilis* infection, injury by a nylon monofilament and a combination of both. The expression level of selected immune-related genes: defensin 1 (Pt*DEF1*), lysozyme 1 (Pt*LYS1*), lysozyme C (Pt*LYSC*), lysozyme M1 (Pt*LYSM1*), autophagy-related protein 101 (Pt*ATG101*), dynamin (Pt*DYN*) and heat shock proteins (HSP70) (Pt*HSPB*, Pt*HSPB2A*, Pt*HSPB2B*), production of lysozyme and HSP70 proteins, and hemocytes viability were measured. The obtained results indicated expression of the lysozyme, autophagy-related protein and HSP70 genes in both ontogenetic stages of *P. tepidariorum*. It has been also shown that the simultaneous action of mechanical and biological factors causes higher level of lysozyme and HSP70, cell apoptosis intensity and lower level of hemocytes viability than in the case of exposure to a single immunostimulant. Moreover, mature females showed stronger early immune responses compared to juveniles.

## Introduction

Spiders are widespread arthropods found in most niches on every continent, excluded Antarctica (Foelix, 2011). Currently, over 50,000 species of spiders are known (World Spider Catalog, 2022), which occupy a wide variety of biotopes and thus—are exposed to many diverse immunostimulants. Spiders at every stage of ontogenesis are constantly exposed to environmental stress. The environmental factors that stimulate the immune system of invertebrates include, among others, injuries (Taylor, Roberts & Uetz, 2006), pathogen infections (Fukuzawa et al., 2008), parasitoid attacks (Schmidt, 2008) or the chemicals such as heavy metals (Wilczek et al., 2018) and pesticides (Laino et al., 2021). Due to such a substantial evolutionary success that the representatives of the Araneae order have achieved, their immune system must be able to respond to various types of immunostimulants and may have mechanisms to counteract the simultaneous action of several immune stimulants at once.

The immune system of spiders is innate and nonspecific. The immune response is based on processes at the cellular and humoral level, mainly based on the reactions of hemocytes. Hemolymph cells are distinguished into a few types: granulocytes, plasmatocytes, cyanocytes, leberidocytes, and prohemocytes—stem cells (Kuhn-Nentwig et al., 2014). The basic types of hemocyte-connected immune responses are phagocytosis, nodulation, melanization, encapsulation, and the production of antibacterial proteins (Kuhn-Nentwig & Nentwig, 2013). In response to the invasion of microorganisms, the hemolymph cells produce antimicrobial proteins, initiate the coagulation cascade, and phagocytize the debris of the eradicated pathogens (Fukuzawa et al., 2008). In addition, invertebrates have a variety of other immunity-related processes, including inflammatory responses and programmed cell death—apoptosis (Smith, 2016).

Apoptosis is a highly controlled reaction of hemocytes to stress induced by physiological and environmental factors, such as pathogens (Hughes et al., 2010). Apoptotic cell death is regulated by various molecules, including heat-shock proteins (HSPs). HSPs are responsible for cytoprotective mechanisms, mainly in controlling protein folding and preventing excessive cell apoptosis (Zugel & Kaufmann, 1999; Carpenter & Hofmann, 2000). Their level may increase in response to high temperature, exposure to heavy metals or pesticides (Köhler et al., 1992), and the reaction to immunostimulation (Srivastava, 2002).

Many proteins produced in invertebrates are involved in immune-related reactions, such as apoptosis, pathogens recognition, tissue growth, hemolymph coagulation, and many other mechanisms (Loker et al., 2004). Among proteins, there is a numerous and specialized group—antimicrobial proteins (AMPs) produced by hemocytes. AMPs are an essential element of the host defense of spiders, widely used to fight bacteria—both G (+) and G (−), but also viruses, protozoa, and fungi. They also have an anti-inflammatory effect (Shin et al., 2020). In general, the principle of antimicrobial peptide action is based on the mechanisms that destroy the structure of the bacterial cell membrane (Lohner, 2009). AMPs are produced in granulocytes, which can store the synthesized peptides in granules (Kuhn-Nentwig & Nentwig, 2013). Due to the continuous circulation of the hemolymph between all spider tissues, antimicrobial peptides can be detected in the hemolymph itself, in venom or organs located in the spider’s abdomen. The most significant number of blood vessels, including the heart, and thus the largest volume of hemolymph in which AMPs are located, is in abdomen (Yiğit & Benli, 2008; Zhao et al., 2011; Rádai et al., 2019).

One of the most widespread AMPs in vertebrates and invertebrates is lysozyme (muramidase), capable of lytic degradation of bacterial membranes. It is a 14.4 kDa enzyme that has antimicrobial activity primarily against Gram (+) bacteria but also against Gram (−) bacteria, viruses, and fungi (Van Herreweghe & Michiels, 2012). Lysozyme has been detected in various spider tissues—in digestive fluids from adult spiders of *Stegodyphus mimosarum* species (Walter et al., 2017), as well as in homogenates from abdomens of *Pardosa agrestis* spiders (Rádai et al., 2019). In addition to lysozymes, defensins are another large family of AMPs in invertebrates. Defensins are small peptides (3–5 kDa) involved in killing pathogens, mainly G (+) bacteria (Schmitt et al., 2010).

Spiders are exposed to various microbes living on the body surface or inside the spider’s prey (Nentwig, 1987; Gemeno, Yeargan & Haynes, 2000). One of the triggers of immune responses is microorganisms, including Gram (+) and Gram (−) bacteria. Bacteria are ubiquitous—they occur in the soil, water, air, and on all surfaces. Therefore every organism is constantly exposed to them (Pickup, 1991). In response to an immunostimulation with G (+) bacteria, an increase in the number of cisterns in the rough endoplasmic reticulum was shown in *Steatoda grossa* granulocytes, which may be associated with the intensified synthesis and storage of proteins involved in immune responses in tested spiders (Wiśniewska et al., 2021).

Knowledge of the spider’s immune system development is very limited so far, although significant differences in immune responses have already been demonstrated in juveniles and adults (Rádai et al., 2019; Kirschman, Dewey & Gregory, 2021). According to current knowledge, age-dependent immune system function may be related to the allocation of energy and nutritional resources, depending on reproductive strategy. Semelparous animals, *i.e*., those that reproduce once in a lifetime, maximize the body’s resources allocated to the reproduction process. Iteroparous organisms, reproducing many times during their lifetime, have to incur a greater metabolic cost associated with keeping the soma in good condition (Lochmiller & Deerenberg, 2000). In the case of the semelparous spider *Pardosa agrestis*, it was proved that subadult individuals showed a more remarkable ability to lyse *Micrococcus luteus* bacterial cell wall than adult spiders. Moreover, differences were also detected between virgin females and females after mating, with unfertilized spiders showing stronger antibacterial responses. This suggests that younger individuals invest many of the body’s resources in efficient host defense, while adults of *P. agrestis* mainly invest in reproduction (Rádai et al., 2019). Trade-offs between reproduction and immunity responses have also been studied in semelparous spider *Tigrosa georgicola* and it was shown that the encapsulation response was not affected by either reproductive state or reproductive effort of the tested females (Kirschman, Dewey & Gregory, 2021). Investing in the maintenance of the female’s soma will be more significant the longer she takes care of her offspring.

Our goal was to check how the immune system is reorganized over the life stages of *Parasteatoda tepidariorum* and how different types of immunostimulation influence the production of selected immunity markers in juvenile and adult individuals. Comparing the immunocompetence between these two ontogenetic stages will allow checking how spiders invest the organism’s resources at different stages of life.

The aim of this study is to verify the following working hypotheses:

H1. The genes encoding selected immune-related proteins from defensin and lysozyme families, autophagy-related protein 101, dynamin, and heat shock proteins are expressed in *P. tepidariorum* spiders.

H2. Mechanical (foreign body) or joint mechanical and biological (infection) intrusion into the integrity of an organism stimulates early immune responses by altering HSP70, lysozyme, apoptotic cell levels and hemocytes viability. The response reflects the intensity of the threat and is stronger the more complex the stimulation.

H3. Immunological potential (gene expression) and reactions (lysozyme and HSP70 production, viability of hemocytes) caused by mechanical damage and bacterial infection have a different course in immature and mature females of *P. tepidariorum*.

## Materials and Methods

### Materials

*Parasteatoda tepidariorum* (formerly: *Achaearanea tepidariorum*) C. L. Koch, 1841 (Araneae, Theridiidae) were obtained from laboratory-bred strains of the Institute of Biology, Biotechnology and Environmental Protection (formerly Department of Animal Physiology and Ecotoxicology), University of Silesia, Poland. The animals were bred at 25 ± 1 °C at 70% relative humidity in a long-day photoperiodic conditions L: 16 h, D: 8 h. Juveniles and adults were fed twice a week *ad libitum* with laboratory cultured *Drosophila melanogaster* or *D. hydei* and were water sprinkled regularly.

For the presented study, the following ontogenetic stages of *P. tepidariorum* were selected for every experimental group:

a) juvenile females—the penultimate nymphal stage, spiders with marked sexual dimorphism, in 30^th^ day after leaving the cocoon, sexually immature,
b) adult females—young, in 45^th^ day after leaving the cocoon, sexually mature, unfertilized individuals.

The spider development time was counted from leaving the cocoon, according to Miyashita (1987) and behavioral observations as described Sawadro et al. (2019).

### Experimental groups

All studies were conducted on *P. tepidariorum* females in experimental groups with a progressive degree of immunostimulation:

a) control (C)—unexposed spiders,
b) stimulated (S)—with a sterile nylon monofilament inserted into the abdomen,
c) stimulated + infected (S + I)—with a nylon fiber coated with *Bacillus subtilis* suspension, implanted in the abdomen.

### Methods

#### Bacterial strain

*Bacillus subtilis* is a Gram (+) bacterium, commonly found in soil and rhizosphere (Stein, 2005). Bacteria of the genus *Bacillus* were detected on the body and chelicerae surfaces of the *Steatoda nobilis*, *Amaurobius similis* and *Eratigena atrica* spiders (Dunbar et al., 2020). As assumed by Gaver-Wainwright et al. (2011), some bacteria species of the genus *Bacillus* spp. may be obligate or opportunistic pathogens for *Tegenaria agrestis* spiders. In studies carried out on *Pardosa astrigera* spiders, antimicrobial peptides—lycotoxins—have been shown to inhibit the growth of the colony-forming units (CFU) of bacteria belonging to the genus *Bacillus* (Shin et al., 2020). Using the multi-task learning method, it has also been proven that the AMPs contained in *Pardosa astriger* venom have a strong antimicrobial effect against five bacterial strains, including *B. subtilis* (Lee et al., 2022). Furthermore, the antibacterial effects of lysozyme against bacteria of the genus *Bacillus* have been proven (Abdou et al., 2007).

Laboratory culture of a bacterial strain *Bacillus subtilis* ATCC 6633 was maintained using lysogeny broth (LB 2% w/v) culture medium at 28 °C for 24 h at 120 rpm. Bacterial strains were kept as frozen stocks at −80 °C in 10% glycerol. The stocks were inoculated into the LB medium and incubated to reach a proper optical density (OD) value corresponding the number of 85 × 10^6^ colony-forming units (CFU/ml) for bacterial strain.

#### Immunostimulation

Immunomodulating effects may be caused not only by a bacterial infection, but also by a foreign body. Phagocytosis, encapsulation and nodulation processes are a reactions of invertebrates to insertion of a small foreign bodies (Cerenius, Lee & Söderhäll, 2008; Lapointe, Dunphy & Mandato, 2012). According to a study conducted by Wilson-Rich et al. (2018), in *Apis mellifera* immunostimulated with a foreign body, implanted fibers coated with phosphate saline buffer triggered an immune reaction. However, monofilaments covered with highly conserved pathogen-associated molecular patterns (PAMPs) mimicking pathogen infections, induced a stronger immune responses than PBS-coated monofilaments.

**Figure 1:**
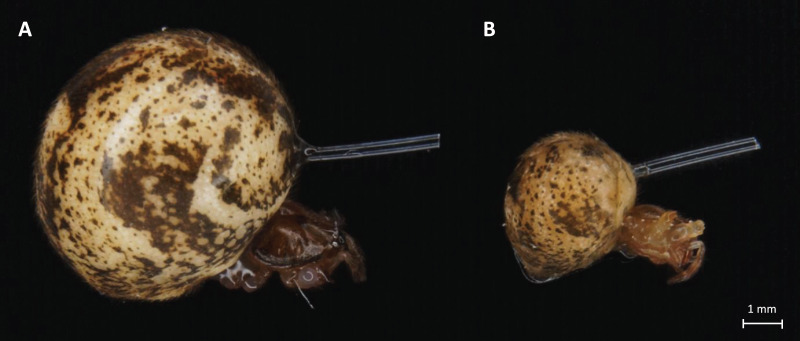
Light microscope images of adult (A) and juvenile (B) *P. tepidariorum* female*s* with nylon fiber in abdomen.

**Figure 2:**
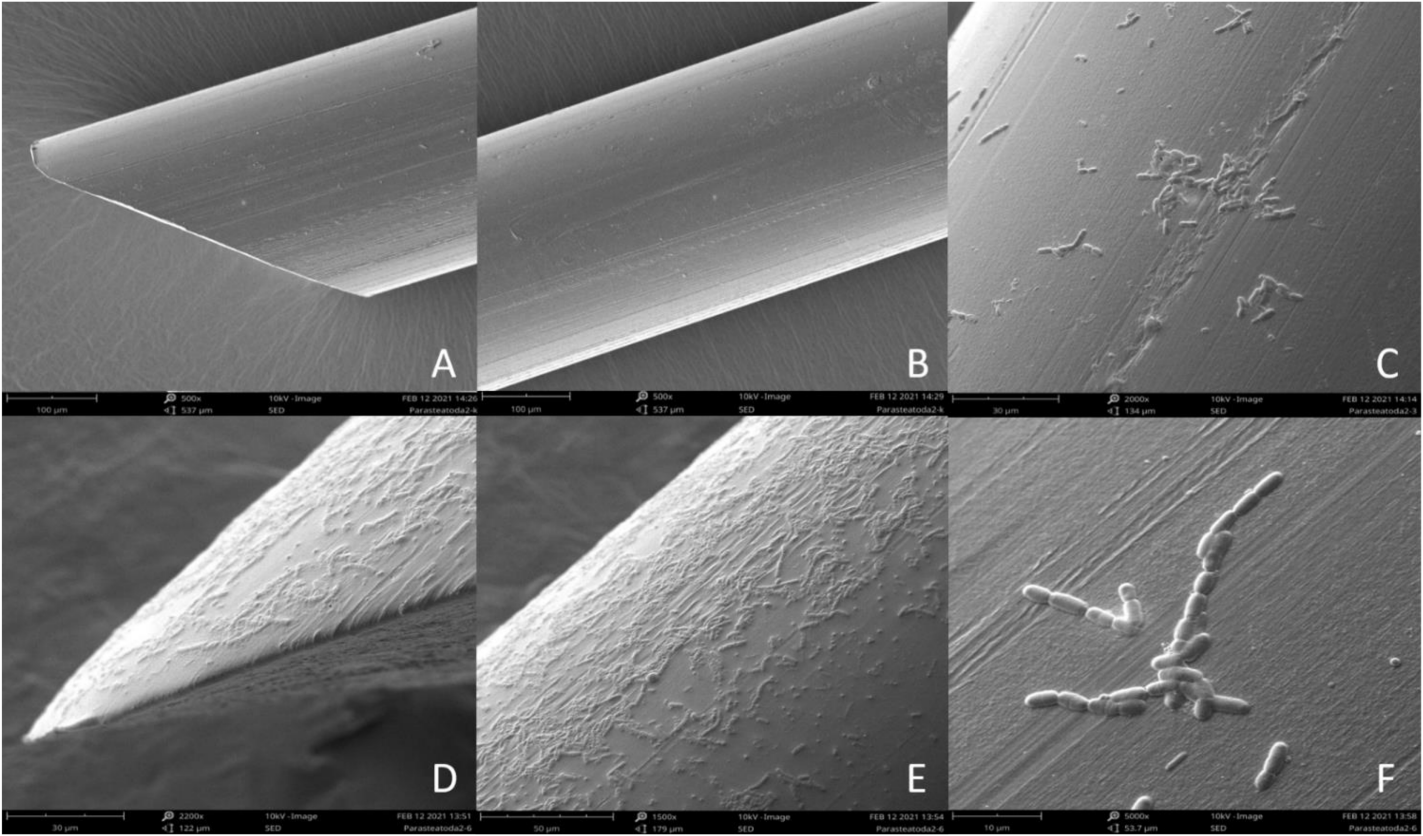
Scanning Electron Microscope (SEM) image of sterile nylon fiber (A and B) and coated with *Bacillus subtilis* bacteria (C–F).

In our experiments, the previous optimization preceded immunostimulation procedures to avoid excessive cases of death or hemolymph loss. The 0.3 mm diameter monofilament fishing line was used to measure the immunostimulation effect. A nylon thread was cut into 4-mm pieces, soaked in 70% ethanol for sterilization, and dried. A part of the prepared thread was soaked in the bacterial suspension for 30 min. Spiders were immobilized on ice and nylon fibers were inserted into their abdomens. The placement site was adapted to both ontogenetic stages of the spiders, based on the survivability and good fitness of both adult and juvenile spiders (shown in Fig. 1). In the stimulated + infected spiders group, the insertion was made with a fiber previously soaked in a suspension of *B. subtilis* (Figs. 2C–2E) but in the stimulated female’s group, only a sterile thread was used (Figs. 2A and 2B). After 1 h, the dissected abdomen of each individual was placed in the 1.5 ml eppendorf tube and used for experiments. Spiders from the control groups were removed from their breeding containers and placed in the new sterile ones for 1 h to maintain the same handling stress as in the experimental groups.

#### Scanning electron microscope

Imaging of the bacterial film present on the surface of the nylon monofilaments used in this study was performed with Hitachi UHR FE-SEM SU 8010 scanning electron microscope with an acceleration of 10 kV using a secondary electron detector (SED). Samples of a sterile nylon fibers and fibers immersed in 85 × 10^6^ CFU/ml solution of *B. subtilis* were placed on aluminum stubs with adhesive carbon tape and allowed to dry completely at 22 °C for 12 h. After this time, the prepared material was covered with a 0.4 mm layer of gold in a Pelco SC-6 sputter coater (Ted Pella Inc., Redding, CA, USA) and observed in a scanning electron microscope.

#### PCR

The expression of the defensin 1 (Pt*DEF1*), lysozyme 1 (Pt*LYS1*), lysozyme C (Pt*LYSC*), lysozyme M1 (Pt*LYSM1*), autophagy-related protein 101 (Pt*ATG101*), dynamin (Pt*DYN*) and heat shock proteins (HSP70) (Pt*HSPB*, Pt*HSPB2A*, Pt*HSPB2B*) genes were studied by the PCR reaction analysis. Data were collected as previously described in Sawadro et al. (2019).

Total RNA from the midgut glands of *P. tepidariorum* juvenile and adult females was isolated using the RNeasy Micro Kit (Qiagen, Hilden, Germany). Previously, spiders were anesthetized at −20 °C and the tissues were dissected on ice in a sterile condition with sterile phosphate-buffered saline (PBS, 137 mM NaCl, 10 mM phosphate buffer (K2HPO4, KH2PO4), 2.7 mM KCl, pH 7.4). All tissues were immediately frozen in liquid nitrogen and stored at −70 °C in the sterile Eppendorf tubes until use.

TURBO DNA-*free* kit^TM^ (Invitrogen, Carlsbad, CA, USA) was used to remove the remaining genomic DNA contamination. The amount of obtained RNA and its quality were estimated using a NanoDrop 2000 spectrometer (Thermo Scientific, Wilmington, DE, USA) and electrophoresis. Using the Reverse Transcription System (Promega, Madison, WI, USA) and random primers, a 1 µg volumes of total RNA was retrotranscribed. The synthesized cDNA was used as a template for PCR.

The PCR reaction was conducted using the PPP Master Mix (Top-Bio, Vestec, Czech Republic). Reactions were performed in 25 μl total volume with 4 ng DNA, 2.5 U Taq Purple DNA polymerase and 20 mM each of the primers under the following thermal conditions: 95 °C for 5 min, 36 cycles at 95 °C for 30 s, 55 °C for 35 s, 72 °C for 45 s and termination at 72 °C for 10 min. Each sample had six biological replicates (*n* = 6) and three technical repetitions. Amplification products were separated by size by the agarose gel electrophoresis. Data analysis relied on confirming the presence of a band at the appropriate height on the agarose gel.

Primers for immune-related genes (Table 1) were designed with available online Primer3Plus software (https://primer3plus.com/cgi-bin/dev/primer3plus.cgi) based on the sequences available in the NCBI (XM_016066117, XM_016074084, XM_016051009.2, XM_016047665.1, XM_016058609.3, XM_043043671.1, XM_016066346.2, XM_016048359.3, XM_016074530.3).

**Table 1:** The forward and reverse sequences for the primers.

Target genes	Forward primer sequence	Reverse primer sequence	Product size (bp)
Defensin 1	TTCACGCAAATTGTGGGGAA	AGCAATCAGAGCAATTGGAATTGA	430
Lysozyme 1	TCTAGCCCCCGGAGCTATTT	CAGGCTCCGAATCCCAACTT	533
Lysozyme C	GCTGCCAAATGGGTATGTCT	CTGGCCATTGTTTGAATCCT	211
Lysozyme M1	ACGCCAAAAGATGGTGAGTC	TGCTGGTAGCTCATCTGGTG	207
Autophagy-related protein 101	GAGGTAGATGGGCTGCTGT	GCCAGGTATCTTTGGCACAT	197
Dynamin	ACATTGAGGGTTCTGGTTCG	TGGATGTTTCGAATCGCATA	163
Heat shock protein beta	AATGCCTTTTGAACGTCGTC	CGAGGTGTTTCTCGATCCAT	159
Heat shock protein 70 B2A	GGTGTCCCACAGATCGAAGT	CCTGCTTCGCAGAATACACA	247
Heat shock protein 70 B2B	TATGGTGCAGCTGTTCAAGC	GCAAGGAATGCGAGAGTTTC	156

#### ELISA

Lysozyme and HSP70 concentration in the midgut glands of the female spiders was measured using Enzyme-Linked Immunosorbent Assay (ELISA) according to standard protocol (Crowther, 2009) optimized for invertebrate according to Kafel et al. (2012), Tarnawska et al. (2019), including standardization for spider samples (Wilczek, Babczyńska & Wilczek, 2013). The homogenates used for analysis were prepared as follows: midgut glands were collected on ice from cut abdomens, using sterile preparation tools. Tissues were placed in a 1.5 ml eppendorf tubes in 100 μl of a 0.1 M PBS buffer (pH 7.4). Next, every sample was homogenized and centrifuged (10,000×*g*, 10 min, 4 °C). The submitochondrial fraction was transferred into a new 1.5 ml microcentrifuge tube and stored at −70 °C until measurements. Before analyses, the total protein concentration in every sample was determined according to Bradford method (1976) using bovine serum albumin (BSA, protein content >95%, Fluka) as the standard. Samples were diluted to the same protein concentration using a PBS buffer, pH 7.4. The ELISA procedure was started by placing 100 µl of samples in wells of the microtiter 96-well Greiner® plate and it was incubated for 16 h at 4 °C. Then the wells were rinsed three times with 0.05% PBS-Tween20 solution. The next step was to fill the wells with 100 µl 1% BSA solution and incubate 1 h at 37 °C. After another triple washing 100 µl of the rabbit anti-lysozyme primary antibody (Abcam, Cambridge, United Kingdom) in 1:5,000 solution or the mouse anti-HSP70 antibody (Sigma-Aldrich, St. Louis, MO, USA) in 1: 1,000 solution were added. Incubation with primary antibodies lasted 2.5 h at 37 °C. The wells were washed three times with 0.05% Tween20 in PBS. A solution of a secondary antibody conjugated with alkaline phosphatase (1: 1,000, goat anti-mouse IgG, pAb, AP conjugate, Bio-Rad, CA, USA) was added. The incubation lasted 2 h at 37 °C. The wells were rinsed three times again. A color reaction buffer was prepared as follow: para-nitrophenyl phosphate dye (pNpp, Phosphatase substrate, Sigma-Aldrich, St. Louis, MO, USA) was added to the diethanolamine buffer (pH 9.5), with a ratio of 1 mg dye per 1 ml of buffer. A total of 100 µl of the prepared solution was added to the wells and incubated 30 min at room temperature. After this time, the absorbance of the samples was measured in a Tecan Infinite M200 Microplate reader at 405 nm. The same procedure for the ELISA assay was used for the series of standard lysozyme and HSP70 solutions. The concentration of lysozyme and HSP70 was then expressed as µg/mg of total proteins.

#### Hemocytes counting

To count the live and dead hemocytes in the experimental groups, 1 h after immunostimulation, the hemolymph in a volume of about 10 µl was collected from each adult spider. Hemolymph, obtained from the hemolymph leakage where the legs were cut off, was collected with an automatic pipette with sterile tips and used for research immediately after collection. The hemolymph was mixed in a 1:2 ratio with 0.4% liquid Trypan blue solution prepared in 0.81% sodium chloride and 0.06% potassium phosphate. Then 10 µl of the obtained mixture was introduced into the Neubauer counting chamber. Hemocytes visible in the counting grid was summed up from 16 squares with dimensions of 25 µm × 25 µm. In the case of juvenile individuals, due to the much smaller volume of hemolymph in the body compared to adults, hemolymph from 4–5 individuals was pooled.

#### Apoptosis

The quantitative analysis of live, apoptotic and dead cells in the hemolymph was measured by MUSE® Cell analyzer (Millipore, Billerica, MA, USA) flow cytometer with 2,000 events. Hemolymph was collected as described above and gently mixed with 0.1 M PBS (pH 7.4) in order not to damage the hemolymph cells and to obtain the cell suspension. According to the manufacturer’s recommendations, measurements were taken using the Muse® Annexin V & Dead Cell Kit (Luminex Corporation, Austin, TX, USA). Measurements were performed after mixing 100 µl of cell suspension and 100 µL of Muse Annexin V & Dead Cell Reagent incubated for 20 min at room temperature in the dark.

### Statistics

All assays for statistical analysis for every method were based on six replicates, performed in duplicate. Measurements were performed from separate spider pools for each method. In total, 90 individuals were used. The results are presented as mean ± SD values. Kolmogorov–Smirnov test was used to check the normality of the distribution and Levene’s test was used to test the equality of variance. If a significant difference was detected, the Tukey’s range test was used for a *post hoc* two-way analysis of variance (ANOVA). Therefore, for ELISA test results, the Pearson correlation coefficient for these parameters was performed in relation to the immunostimulation degree (control, stimulated, stimulated + infected). For each test, the significance level was 0.05. The results and graphs were analysed in GraphPad Prism® version 9 software (GraphPad, San Diego, CA, USA).

## Results

### PCR

**Figure 3:**
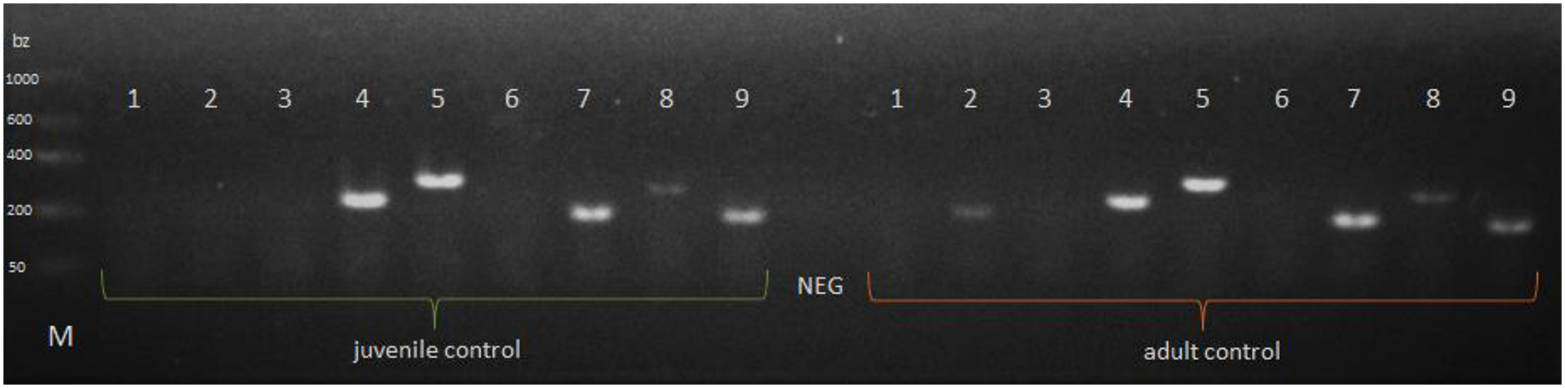
Expression of a selected genes in *P. tepidariorum* spiders at control of various age groups. NEG—negative control without any nucleic acid, M—molecular-weight size marker. *P. tepidariorum* spider ontogenesis stage: juvenile and adult. Detected genes: Pt*DEF1* (1)—defensins, Pt*LYS1* (2), Pt*LYSC* (3), Pt*LYSM1* (4)—lysozymes, Pt*ATG101* (5)—autophagy-related protein 101, Pt*DYN* (6)—dynamin, Pt*HSPB* (7), Pt*HSPB2A* (8), Pt*HSPB2B* (9)—heat shock proteins.

The *P. tepidariorum* M1-type lysozyme (Pt*LYSM1*) and autophagy-related peptide 101 (Pt*ATG101*) were explicitly expressed in the extract from the midgut glands of *P. tepidariorum* juvenile and adult females. The presence of the Pt*HSPB*, Pt*HSPB2A*, Pt*HSPB2B* transcript was also observed in both female age groups, whereas expression of the 1-type lysozyme (Pt*LYS1*) gene was confirmed only in adults. The presence of the Pt*DEF1*, Pt*LYSC*, and Pt*DYN* transcripts was not confirmed (Fig. 3).

### ELISA

**Figure 4:**
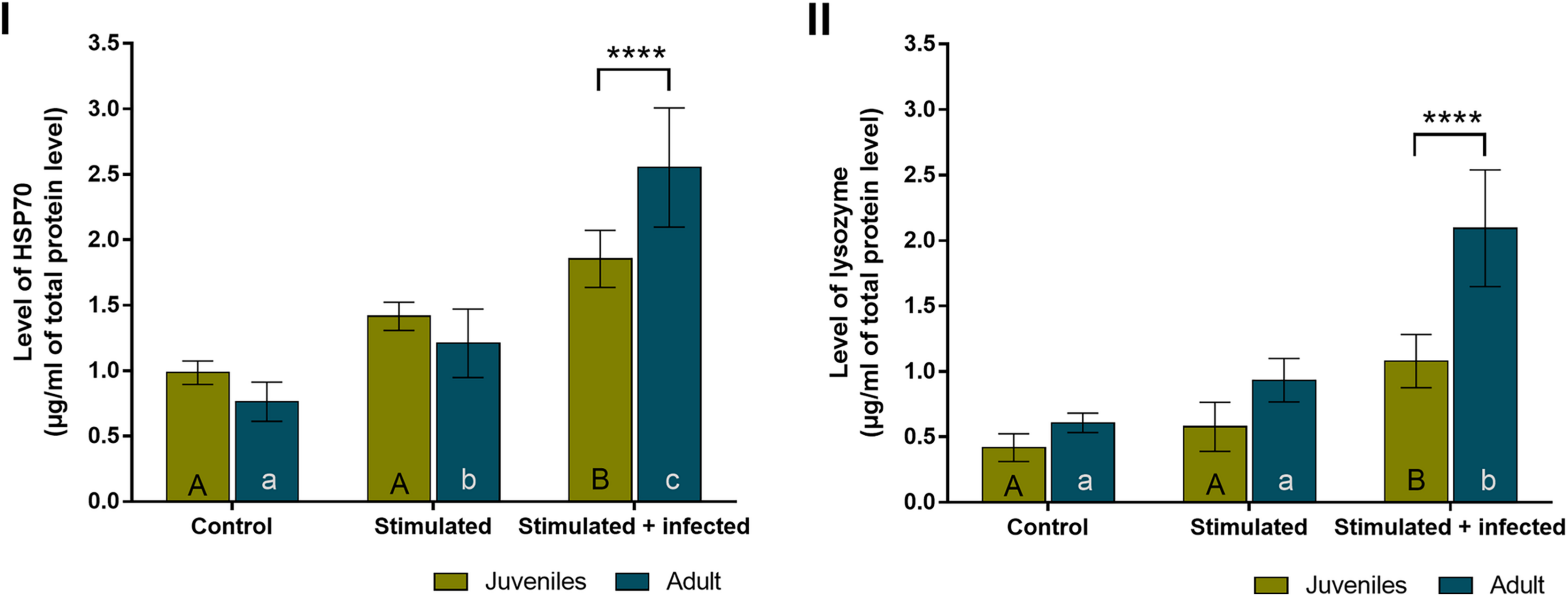
Levels (mean + SD) of HSP70 (A) and lysozyme (B) in *P. tepidariorum* adult and juvenile females after immunostimulation. Tuckey’s multiply comparison test, *p* ≤ 0.05. Different letters indicate statistically significant differences: within immunostimulation (control, stimulated, stimulated + infected), degrees in juveniles (uppercase letters) and adult spiders (lowercase letters). Asterisks indicate statistically significant differences between age groups (adult and juveniles spiders)—(****) *p* < 0.0001.

In all experimental groups of spiders, the overall level of HSP70 (Fig. 4A) was almost twice higher than the level of lysozyme (Fig. 4B). The levels of both proteins did not show statistically significant age-dependent differences within the control group and the stimulated group. The level of HSP70 was significantly higher during the immunostimulation in subsequent degrees only in adult females. Moreover, the HSP70 concentration in the stimulated + infected group was significantly higher than in the stimulated group. In the stimulated groups, the HSP70 level was higher by approximately 1.5 times in juveniles and adult spiders than in the control groups. In the stimulated + infected groups, there were 2.5 times higher levels of this protein in juveniles and 3.5 times higher levels in adult females of *P. tepidariorum*. In addition, in groups where *Bacillus* bacteria were used, a significant difference was found between juveniles and adults (Fig. 4A, Table 2). On the other hand, the level of lysozyme was significantly higher only in the stimulated + infected group in both juveniles and adults. In the juveniles, two times higher lysozyme level and in adults four times higher level in the stimulated + infected group compared to control was noted. However, in the stimulated + infected groups, lysozyme levels were significantly higher in adult females relative to juveniles (Fig. 4B, Table 3). Furthermore, the levels of HSP70 and lysozyme are not correlated with each other in juveniles (Pearson correlation coefficient, r = 0.9597; *p* = 0.1813), while in adults, it shows a high positive correlation coefficient (Pearson correlation coefficient, r = 0.9995; *p* = 0.0208).

**Table 2:** Analysis of variance (ANOVA) of the heat-shock proteins 70 level. F = 13.67, df = 30. Data marked in red indicates a statistically significant *p*-value (*p* ≤ 0.05).

	C. juv.	C. adult	S. juv.	S. adult	S + I juv.	S + I adult
C. juv.	X	0.6299	0.0518	X	<0.0001	X
C. adult		X	X	0.0398	X	<0.0001
S. juv.			X	0.7000	0.0458	X
S. adult				X	X	<0.0001
S + I juv.					X	0.0004
S + I adult						X

**Table 3:** Analysis of variance (ANOVA) of the lysozyme level. F = 10.70, df = 30. Data marked in red indicates a statistically significant *p*-value (*p* ≤ 0.05).

	C. juv.	C. adult	S. juv.	S. adult	S + I juv.	S + I adult
C. juv.	X	0.7104	0.8323	X	0.0003	X
C. adult		X	X	0.1739	X	<0.0001
S. juv.			X	0.1118	0.0085	X
S. adult				X	X	<0.0001
S + I juv.					X	<0.0001
S + I adult						X

### Hemocytes counting

**Figure 5:**
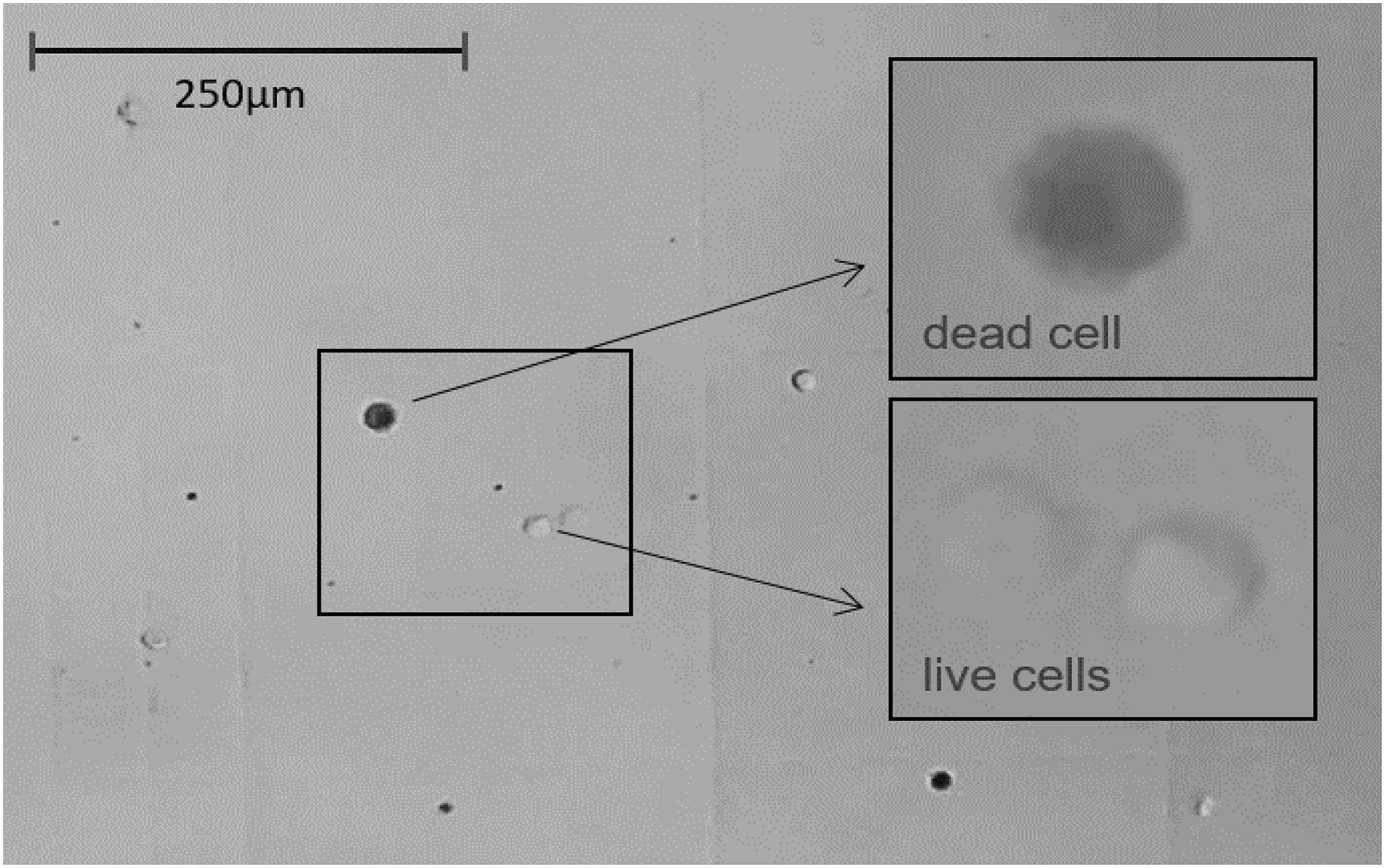
The light microscope view (20×) to Neubauer counting chamber. Hemocytes collected immediately after 1 h of immunostimulation was stained with Trypan blue solution 0.4% and counted. Average number of cells/ml of hemolymph ≈ 250,000. Average hemolymph cell size ≈ 13 μm ⌀.

The results obtained with the hemocytometer showed that the immunostimulated spiders were characterized by a higher number of dead hemolymph cells than the control groups. The percentage of dead hemocytes was the highest in the stimulated + infected experimental groups. The tested spiders had a foreign body stuck in their abdomen with simultaneous exposure to *B. subtilis* infection (Fig. 5, Table 4).

**Table 4:** Analysis of variance (ANOVA) of the hemocytes viability measured in Neubauer hemocytometer. F = 75.79, df = 30. Data marked in red indicates a statistically significant *p*-value (*p* ≤ 0.05).

	C. juv.	C. adult	S. juv.	S. adult	S + I juv.	S + I adult
C. juv.	X	<0.0001	<0.0001	X	<0.0001	X
C. adult		X	X	<0.0001	X	<0.0001
S. juv.			X	<0.0001	<0.0001	X
S. adult				X	X	<0.0001
S + I juv.					X	<0.0001
S + I adult						X

**Figure 6:**
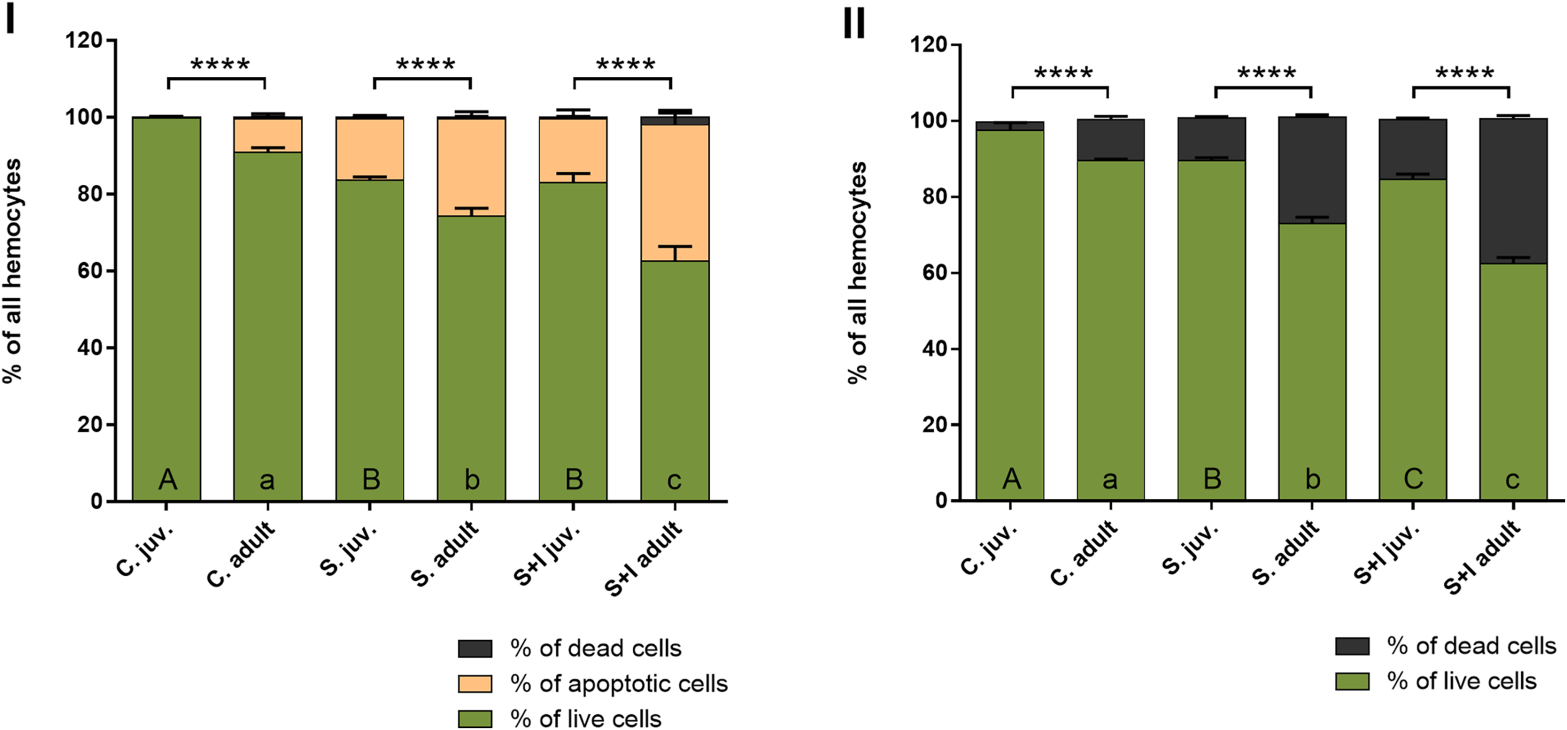
Viability of hemocytes in the hemolymph of *P. tepidariorum* adult females and juveniles exposed to immunostimulation, measured in Muse cytometer (I) and in Neubauer hemocytometer (II). Tuckey’s multiply comparison test, *p* ≤ 0.05. Different letters indicate statistically significant differences: within immunostimulation (control, stimulated, stimulated + infected) degrees in juveniles (uppercase letters) and adult spiders (lowercase letters). Asterisks indicate statistically significant differences between age groups (adult and juveniles spiders)—(****) *p* < 0.0001.

The results obtained with the Muse® Annexin V & Dead Cell Kit, in contrast to the hemocytes counting with the Neubauer chamber, make it possible to distinguish the stages of cell death. The obtained results show that in the case of adult females, the percentage of hemolymph cells, both apoptotic and dead, is getting higher with the advancement of immunostimulation. In juvenile spiders, statistically significant differences between the percentage of live and non-live cells were found only within the control group and the groups exposed to immunostimulants (Fig. 6, Table 5).

**Table 5:** Analysis of variance (ANOVA) of the hemocytes viability measured in Muse cytometer. F = 26.84, df = 30. Data marked in red indicates a statistically significant *p*-value (*p* ≤ 0.05).

	C. juv.	C. adult	S. juv.	S. adult	S + I juv.	S + I adult
C. juv.	X	<0.0001	<0.0001	X	<0.0001	X
C. adult		X	X	<0.0001	X	<0.0001
S. juv.			X	<0.0001	0.9154	X
S. adult				X	X	<0.0001
S+I juv.					X	<0.0001
S+I adult						X

## Discussion

There is a knowledge gap about the development and functioning of the spider’s immune system at different stages of their ontogenesis. While several studies have been carried out to elucidate the antimicrobial properties of spider’s venom, little data exists about the immune responses of the hemocytes themselves. Spider immunity is innate and, therefore, nonspecific—it relies on general universal mechanisms against many pathogens.

Thanks to an efficient immune system, it is possible to fight infection and maintain homeostasis through various humoral and cellular reactions (Romo, Pérez-Martínez & Castillo Ferrer, 2016). Humoral-type immune reactions include, among others, the production of antimicrobial proteins, phenoloxidase (PO) and other enzymes belonging to the cascades that induce a melanization or coagulation of the hemolymph. However, in the case of cellular immunity, there are hemocyte-dependent reactions, such as phagocytosis or encapsulation. Both types of immune responses occur in the body simultaneously, and the combination of their activities enables the effective combating of pathogens and efficient repair of the effects of heavy metals, mechanical damage, or other stressors (Lavine & Strand, 2002).

It is known that the genes encoding some antibacterial proteins are constitutively expressed. In the early stage of the immune reaction, there are immediate reactions involving the migration of hemocytes to the site of the appearance of the immunostimulant and, during the phagocytosis process, the release of antimicrobial proteins stored in hemocyte granules. This is followed by the release of AMPs into the extracellular fluid. Subsequently, late immune responses involving the whole organism occur after about 24 h (Mitta et al., 2000a, 2000b; Mitta, Vandenbulcke & Roch, 2000). It was also confirmed that environmental stress could cause changes in the transcriptome and gene expression profile. The influence of heavy metals, including cadmium (Cd), is already known on the expression of selected immune-related genes in spiders and changes in protective enzyme activity. In the case of *Pirata subpiraticus* spiders, transcriptome analysis showed that Cd exposure causes, among others, changes in the energy metabolism of proteins and fats, in the level of peroxidase activation, as well as in the expression of enzymes related to apoptosis. These changes were time-depend and concentration-depend (Yang et al., 2021). Also, Yang et al. (2018) showed that exposure to Cd has an effect on venom glands immunology in wolf spiders *Pardosa pseudoannulata*. Changes in the expression of immune-related genes, including those involved in apoptosis, platelet activation, or bacterial invasion of epithelial cells, have been demonstrated. The study of the *P. pseudoannulata* transcriptome also showed that the change in gene expression after exposure to cadmium causes a decrease in the immune function of the venom glands (Lv et al., 2021). Thus, exposure to environmental stressors, such as heavy metals, can alter the expression of genes related to the immune system and affect the detoxification processes of spiders.

To initially assess the immunocompetency of *P. tepidariorum*, in our research, the following genes encoding proteins responsible for reactions related to the spider’s immune system were selected for PCR detection: defensins (Pt*DEF1*), lysozymes (Pt*LYS1, P*t*LYSC*, Pt*LYSM1*), autophagy-related protein (Pt*ATG101*), dynamin (Pt*DYN*) and heat shock proteins (Pt*HSPB*, Pt*HSPB2A*, Pt*HSPB2B*). Our results showed a dependence of gene expression on the age of the spiders and suggest that genes differentially expressed between juveniles and adults may be functionally involved in immune function and stress response. Age-dependent changes in gene expression may be associated with differences in physiological needs and energy metabolism between the younger and older organism as previously described *i.e*., in *D. melanogaster* (Kim et al., 2005; Bordet et al., 2021).

From among the immune-related genes tested in this study, we detected the presence of genes encoding lysozymes—Pt*LYSM1* and Pt*LYS1*, while Pt*LYS1* is expressed only in adult spiders (Fig. 4). Apart from genes encoding typically antimicrobial compounds like defensins and lysozymes, we confirmed the expression of an autophagy-related and heat shock proteins encoding genes in both tested development stages of the *P. tepidariorum* spiders. Autophagy-related proteins (ATG) take part in the subsequent stages of the autophagy reaction: from initiation, nucleation of the membrane, phagosphore formation and elongation, lysosome fusion, ending with a substrate degradation in autolysosome. These proteins are a large family of molecules influencing immunocompetence and have already been detected in other invertebrates. ATG101 protein in *D. melanogaster* is involved in phagosome formation and ultimately may lead to pathogen elimination (Kuo, Hansen & Troemel, 2018). Moreover, in the case of other arachnids (ticks), it has been proven that genes from the ATG group are highly conserved. Some of them are already expressed in the earliest ontogenetic stages (eggs, larvae) of *Ixodes scopularis* (Wang et al., 2020). Another tested group of genes encoding immune-related proteins in PCR were heat shock proteins. They correspond to adaptation to a wide range of stressors—from elevated temperature, through UV radiation, to pathogen infection (Feder & Hofmann, 1999). Expression of genes encoding proteins from the HSP family has been demonstrated in invertebrates closely related to spiders: the HSP70 gene in *Dermatophagoides farinae* tick (Yang et al., 2019) and the HSP90 gene in the spider mite *Tetranyus cinnabarinus* (Feng et al., 2010). In the *Dermacentor silvarum* tick, the expression of both HSP70 and HSP90 genes was demonstrated, and the high conservative nature of these genes was also confirmed (Agwunobi et al., 2021). In addition, the expression of genes encoding proteins from the HSP family, including two from the HSP70 group, was detected in the spider *Pardosa pseudoannulata* (Xiao et al., 2016). In the case of the studies of the *P. tepidariorum* spiders, our results showed the expression of the Pt*HSPB*, Pt*HSPB2A* and Pt*HSPB2B* genes in both adult and juvenile individuals. In contrast, expression of the gene encoding dynamine—a protein involved in endocytosis—was not demonstrated in tested spiders. In the case of other invertebrates, a test has been performed to confirm the expression of the gene encoding dynamine orthologue in *D. melanogaster* fly (Gonzalez-Bellido et al., 2009), while the presence of dynamin 1 itself has not been found outside of mammalian lineage (Suyama, 2013). Based on our preliminary assessment of the immune potential carried out by the PCR method, it was found that *P. tepidariorum* spiders showed expression of selected genes encoding immune-related proteins. The spiders from this species possess a constitutive immunological potential based on antimicrobial proteins, including representatives of HSP and lysozymes in further parts of this study. Moreover, we cannot exclude as well the expression of other AMPs not tested in this study. Therefore the working hypothesis of H1 was confirmed in relation to some of the tested genes.

The next step of the present study was to check if the immune system of the tested juvenile and adult *P. tepidariorum* spiders is capable of activating the internal defense processes. So far, the constitutive production of AMPs and their storage in hemocyte granules has been confirmed in *Acanthoscurria gomesiana* (Fukuzawa et al., 2008). While Ali et al. (2021) tested the antibacterial activity of invertebrate lysates, including the prosoma and opisthosoma of spider *Grammostola rosea*, against Gram (−) *Escherichia coli* K1 and Gram (+) methicillin-resistant *Staphylococcus aereus* (MRSA). Both parts of the spider’s body showed antibacterial activity after 2 h of incubation with bacterial suspension at a concentration of 10^8^ CFU/mL. The lysates more strongly inhibited bacterial growth in the case of MRSA. For other arthropods—*Gryllus texensis* crickets, hemolymph-PBS mixture was mixed with *Micrococcus luteus* cell wall suspension and lysozyme-like activity was measured. The lytic activity of the hemolymph increased after 30 and 60 min of measurement, and after 24 h, the level returned to the control level (Adamo, 2004).

Our findings indicated that both juvenile and adult *P. tepidariorum* spiders are capable of immune response to biotic and abiotic targets. Therefore, hypothesis H2 can be accepted. In females exposed to immunostimulation with the inserted sterile nylon monofilament and for the combination of the inserted fiber with simultaneous bacterial infection, higher level of HSP70 were demonstrated compared to the control group. Additionally, the level of HSP70 increased in adult spiders of the species *Pardosa lugubris* under stress conditions. Therefore it is assumed that this protein may have the function of preventing excessive apoptosis intensification (Wilczek, 2005). In our investigation, the levels of both studied proteins were characterized by an increasing trend along with the advancement of the degree of immunostimulation. There were also statistically significant differences in the level of HSP70 between juvenile and adult females in the stimulated + infected group and between the level of lysozyme in both experimental groups. In all these cases, adult spiders were characterized by a higher level of the studied proteins. Also, in both experimental groups, adult females showed stronger apoptotic responses than juvenile ones.

The level of HSP70 and lysozyme was not correlated in juveniles, while it showed a high positive correlation coefficient in adults. However, the correlation between the level of HSP70 and lysozyme was found only in adults, in juveniles, such a relationship has not been revealed. In *P. tepidariorum* adults, a correlation between the level of lysozyme and HSP70 has been demonstrated, as both proteins participate in immune-related reactions.

All the results obtained in the present study indicate stronger immune responses in adult *P. tepidariorum*. They are most likely related to the fact that in mature spiders, the immune system is fully formed, and the energy resources are distributed appropriately between immunity and reproduction. The working hypothesis H3 was also verified as correct. A study conducted on *Drosophila melanogaster* flies proved that crucial mechanisms of survivability are age-dependent. Juveniles tolerate developing infections by controlling the intensity of the immune response and maximizing the amount of resources to maintain readiness for reproduction. In contrast, older individuals are likely to experience trade-offs between reproduction and somatic functions, including immunology. Level of many AMPs increases with age, but older individuals show increasing impairments in immune responses. Reproductive costs are presumably related to the activation of AMPs synthesis since amino acids are used in producing these molecules and, therefore cannot be targeted to egg production (Zerofsky et al., 2005). Moreover, adult spiders do not have to invest resources in energy-intensive moulting processes as in the case of immature individuals (Herzig, 2010; Santana et al., 2017). In a study on the infected wolfspider *Schizocosa ocreata*, juvenile males have been shown to allocate more resources to fighting infection in the case of trade-offs between immunity and sexual development. This results in less mating success in the future. Males in the juvenile stage exposed to bacterial infection as mature individuals showed more intensive encapsulation and melanization density than uninfected males (Gilbert, Karp & Uetz, 2016). Research by Rádai et al. (2019) showed that juvenile individuals in the *P. agrestis* spider were characterized by a higher level of lysozyme than adults. Scientists suggest that this dependence may result from the reproductive characteristics of these spiders and affect the energy costs that are spent differently in subadult and adult individuals. After mating, spiders of the genus *Pardosa* most often form only up to three cocoons with eggs during their life (Dolejš, 2013). Therefore a large part of the energy resources is allocated to the processes related to reproduction and type of parental care, and its length affects the number of resources invested in immune responses (Kirschman, Dewey & Gregory, 2021). Fertilized females of *P. tepidariorum* lay several egg sacs during their lifetime. An adult spider must consequently invest a lot of energy in maintaining homeostasis to effectively produce as many eggs as possible. *P. agrestis* and *P. tepidariorum* spiders have various reproductive strategies, respectively semelparous and iteroparous. Therefore there may be differences in the intensity of immune responses in mature and immature individuals.

An important age-dependent indicator was also the level of apoptosis. Apoptosis is a natural, physiological regulatory process of programmed cell death that controls the organism’s development by removing damaged or abnormal cells (Vaux & Korsmeyer, 1999). It is involved in maintaining homeostasis and responding appropriately to all environmental stressors in both growing and fully mature animals (Agnello et al., 2015). If an apoptotic cell is not effectively and quickly cleared from the body, it can cause an inflammatory reaction (Nagata, Hanayama & Kawane, 2010). Programmed cell death is necessary for the proper formation and maturation of gametes (Nezis et al., 2000) and therefore protects the body against unnecessary energy expenditure on maintaining sex and somatic cells that contain abnormalities and also keeping a balance between the number of both types of cells (Baum, St. George & McCall, 2005). In the case of adults of *Anopheles gambiae* mosquitoes infected with the protozoan *Plasmodium yoelii*, an increase in follicular apoptosis in ovaries was observed and, thus, simultaneously a decrease in the number of produced eggs. There was a 440.9% increase in apoptosis compared to the control group, which once again suggests the existence of trade-offs between reproduction and immunity in invertebrates (Ahmed & Hurd, 2006). During the development of the spider embryo, including germ band inversion, nervous system formation, and leg development, apoptosis is occurred as well. Until about 80 h and about 145 h after oviposition, programmed cell death occurs at a much lower level in juveniles (Prpic & Damen, 2005). Apoptosis also removes cells damaged permanently by the environmental stressor (Agnello & Roccheri, 2010). In our investigation, both mechanical damage and bacterial infection were such stressors. The mechanisms leading to programmed cell death were found in all our experimental groups, which is consistent with the current state of knowledge about the prevalence and conservativeness of apoptosis in the animal world.

## Conclusions

The results obtained in this study showed that spiders immune potential and immune response are age-dependent. It was also shown that *P. tepidariorum* spiders could respond to both abiotic and biotic immunostimulants. Mature *P. tepidariorum* females showed stronger immune responses than juveniles, imply that adults are better adapted to environmental conditions. In addition, the response was greater with the advancement of immunostimulation (foreign body, foreign body + bacterial infection), confirming that the immune system of spiders may have mechanisms to counteract the simultaneous action of several immunostimulants at once. Age-dependent changes may be associated with differences in physiological needs and energy metabolism between the younger and older organism. While, the trade-offs between allocating resources to energy-consuming immune responses and other processes, including reproduction, may vary depending on the spider’s breeding strategy. In this work, it was shown that a wide range of non-specific defense mechanisms, both cellular and humoral, play an important protective role in spiders in response to immunostimulation. To learn about the mechanisms of the understudied arachnid’s defense system, the results of our research are an introduction to the planned extended investigation in the field of spider immunocompetence.

## Supplemental Information

10.7717/peerj.15337/supp-1 Supplemental Information 1 Absorbance values of samples used for the detection of lysozyme and HSP70 levels by ELISA. C—control group, S—group stimulated with sterile nylon fiber, S + I—group stimulated with nylon fiber coated with *Bacillus subtilis* bacteria.

10.7717/peerj.15337/supp-2 Supplemental Information 2 The quantitative analysis of live, early, late apoptosis and cell death in the hemolymph obtained using the Muse® Annexin V & Dead Cell Kit. C—control group, S—group stimulated with sterile nylon fiber, S + I—group stimulated with nylon fiber coated with *Bacillus subtilis* bacteria.

## Additional Information and Declarations

### Data Availability

The following information was supplied regarding data availability: The raw data is available in the Supplemental Files.
